# Loss of Polycomb Repressive Complex 2 Function Alters Digestive Organ Homeostasis and Neuronal Differentiation in Zebrafish

**DOI:** 10.3390/cells10113142

**Published:** 2021-11-12

**Authors:** Ludivine Raby, Pamela Völkel, Shaghayegh Hasanpour, Julien Cicero, Robert-Alain Toillon, Eric Adriaenssens, Isabelle Van Seuningen, Xuefen Le Bourhis, Pierre-Olivier Angrand

**Affiliations:** 1 Univ. Lille, CNRS, Inserm, CHU Lille, UMR 9020-U 1277 – CANTHER – Cancer Heterogeneity Plasticity and Resistance to Therapies, F-59000 Lille, France; ludivine.raby.etu@univ-lille.fr (L.R.); pamela.voelkel@univ-lille.fr (P.V.); julien.cicero.etu@univ-lille.fr (J.C.); robert-alain.toillon@univ-lille.fr (R.-A.T.); eric.adriaenssens@univ-lille.fr (E.A.); isabelle.vanseuningen@inserm.fr (I.V.S.); xuefen.le-bourhis@univ-lille.fr (X.L.B.); 2 Department of Fisheries and Animal Sciences, Faculty of Natural Resources, University of Tehran, Karaj 31587-77871, Iran; shaghayegh.hasanpour88@yahoo.com; 3 Univ. Artois, UR 2465, Laboratoire de la Barrière Hémato-Encéphalique (LBHE), F-62300 Lens, France

**Keywords:** zebrafish, epigenetics, PRC2, EED, polycomb repression

## Abstract

Polycomb repressive complex 2 (PRC2) mediates histone H3K27me3 methylation and the stable transcriptional repression of a number of gene expression programs involved in the control of cellular identity during development and differentiation. Here, we report on the generation and on the characterization of a zebrafish line harboring a null allele of *eed*, a gene coding for an essential component of the PRC2. Homozygous *eed*-deficient mutants present a normal body plan development but display strong defects at the level of the digestive organs, such as reduced size of the pancreas, hepatic steatosis, and a loss of the intestinal structures, to die finally at around 10–12 days post fertilization. In addition, we found that PRC2 loss of function impairs neuronal differentiation in very specific and discrete areas of the brain and increases larval activity in locomotor assays. Our work highlights that zebrafish is a suited model to study human pathologies associated with PRC2 loss of function and H3K27me3 decrease.

## 1. Introduction

Polycomb group (PcG) proteins are epigenetic repressors involved in a variety of biological processes, including stem cell renewal and control of cell identity during differentiation and development [1,2,3]. PcG proteins associate into two main histone-modifying protein complexes named polycomb repressive complexes 1 and 2 (PRC1 and PRC2) [4,5,6]. According to the current canonical model, PRC2 mono-, di-, and trimethylates lysine 27 of histone H3 (H3K27me1/2/3) [7,8,9] leading to the recruitment of PRC1 that in turn monoubiquitinylates lysine 119 of histone H2A (H2AK119ub) [10,11]. These histone modifications induce chromatin compaction and transcriptional silencing at numerous PRC2-target gene expression programs involved in cell fate, cell differentiation, lineage specification, or development [12,13,14,15,16,17,18,19]. Thus, PRC2 that initiates polycomb repression appears central to epigenetic regulation. The PRC2 core complex consists of four stoichiometric factors EZH2 or EZH1, SUZ12, EED, and RBBP4/7. The first three proteins, EZH1/2, SUZ12, and EED, form the minimal complex possessing the histone methyltransferase activity. EZH2 and EZH1 are two mutually exclusive paralogs harboring the catalytic activity, but EZH2 appears to be the main catalytic subunit of PRC2 during development and remains more efficient than EZH1 at methylating H3K27 [20]. However, the catalytic activity of EZH1/2 requires the physical association with EED and SUZ12 as their absence inhibits PRC2 activity. RBBP4/7 is required for PRC2 binding to nucleosomes and enhances its methyltransferase activity. In addition, a number of facultative subunits have been reported to bind to the PRC2 core complex at a substoichiometric level defining at least two distinct multimeric complexes, PRC2.1 and PRC2.2. PRC2.1 contains one of the PCL proteins (PHF1, MTF2, or PHF19), together with EPOP or PALI1, whereas PRC2.2 is associated with AEBP2 and JARID2. These additional proteins are involved in PRC2 recruitment to chromatin, stabilization of the protein complex, and/or modulation of its activity [5,21,22,23]. In this landscape, SUZ12 and EED are the only two components associated with all PRC2 alternate protein complexes and are absolutely required for deposition and spreading of the H3K27me2/3 epigenetic marks into the epigenome. Indeed, these components are essential for early mouse development, and mice lacking Suz12 or Eed functions are not viable and die during early postimplantation stages [24,25]. In humans, EED mutations and variants have been identified in different pathologies, including cancers [26,27,28,29] as well as abnormal developmental syndromes [30,31,32,33,34].

Here, we describe the use of the zebrafish model to investigate the function of Eed. Zebrafish (*Danio rerio*) is a widely used vertebrate model for studying gene functions in development, morphogenesis as well as in physiopathological conditions. Furthermore, this organism is particularly suited to study the role of the PRC2 complex and H3K27me3 methylation. Indeed, it has been previously shown that in contrast to mice, loss of function of several core subunits of the PRC2 complex, including Ezh1 [35,36], Ezh2 [37,38], and Suz12a/b [39], does not lead to zebrafish lethality at gastrulation. Instead, *ezh2* and *suz12a*; *suz12b* homozygous mutants die at around 12 days post fertilization (dpf), after body plan formation and tissue specification [37,38,39], whereas *ezh1* homozygous mutants are viable and fertile [35,36]. This absence of embryonic lethality in zebrafish allows the study of the role of H3K27me3 methylation in tissue development and homeostasis without the requirement of conditional knockout strategies. In this study, we generated and characterized null alleles of *eed* to investigate the role of PRC2 function and H3K27me2/3 methylation in zebrafish development and organogenesis. Homozygous *eed^−/−^* mutants die at around 10–12 dpf with strong defects at the level of the digestive organs, including reduced size of the pancreas, hepatic steatosis, and loss of the intestinal structures. However, this relatively late lethality allowed us to focus on the expression of markers of the developmental brain. We found that PRC2 loss of function alters neuronal differentiation in very specific and discrete areas of the brain. Furthermore, locomotor activity assays highlighted the role of PRC2 in the zebrafish larval behavior. Altogether, our observations showed that this *eed*-deficient zebrafish line could constitute a valuable model to study the impact of H3K27me3 decrease and PRC2 loss of function in development as well as in several human pathologies.

## 2. Materials and Methods

### 2.1. Zebrafish Maintenance, Embryo Preparation, and Animal Ethics Statements

Zebrafish from the TU strain and Tg(actb2:GFP-Hsa.UTRN)^e116^ transgenics [40] are maintained at 27.5 °C in a 14/10 h light/dark cycle. The evening before spawning, males and females were separated into individual breeding tanks (Tecniplast, Decines-Charpieu, France). Spontaneous spawning occurred the following morning when the plastic divider was removed. Embryos were collected and staged according to Kimmel et al. [41]. The chorions were removed from embryos using 1% pronase (Sigma, St. Louis, MO, USA) for 1 min. Zebrafish embryos were fixed overnight in 4% paraformaldehyde in PBS (phosphate-buffered saline), dehydrated gradually to 100% methanol, and stored at −20 °C.

Zebrafish were maintained in compliance with the French and European Union guidelines for the handling of laboratory animals (Directive 2010/63/EU of the European Parliament and of the Council of 22 September 2010 on the protection of animals used for scientific purposes). The experimental procedures carried out on zebrafish were reviewed and approved by the local Ethics Committee, CEEA 75 Nord Pas-de-Calais, and the French Ministry of Higher Education and Research (APAFiS approval number 13527-2018011722529804_v3).

### 2.2. TALEN Design and Assembly

TALENs targeting *eed* were designed and assembled as previously described [42]. The *eed* TALEN target site was selected using the online TAL Effector-Nucleotide Targeter tool (https://tale-nt.cac.cornell.edu/, last accessed on 11 October 2021 [43]) in exon 3 (ENSDART00000162928.1) with the presence of a restriction site (BseNI) within the spacer sequence for screening and genotyping purposes.

Eed-specific TALEN constructs were engineered using the TALEN Golden Gate assembly system described by Cermak et al. [44] using the expression backbones, pCS2TAL3DD and pCS2TAL3RR [45]. Plasmids for Golden Gate Cloning were obtained from Addgene.

### 2.3. mRNA Injection into Zebrafish Embryos

Capped mRNAs were synthetized using SP6 mMESSAGE mMACHINE kit (Ambion) from linearized plasmid templates. mRNAs (50–100 pg) were injected into 1-cell zebrafish embryos using a FemtoJet microinjector (Eppendorf, Matesson, France).

### 2.4. Genotype Analyses

For genotyping, embryos, larvae or caudal biopsies of larvae were incubated in 10 μL PCR extraction buffer (10 mM Tris-HCl pH 8.0, 2 mM EDTA, 0.2% Triton X-100, 100 μg/mL proteinase K) and placed at 50 °C for 4 h prior to proteinase K inactivation at 95 °C for 5 min. Genotype analysis was performed by PCR on 2.5 μL of samples using the primer set TAL_eed_5′ (CAGCCTAAAGGTGAGGCCGC) and TAL_eed_3′a (CGATCTCGACCCGGAACAC) followed by PCR product digestion with the BseNI restriction enzyme. An alternate genotyping strategy consists of the PCR amplification of genomic DNAs with the primer set TAL_eed5_new GTGTGCAGGAGGATCATGGTCAG) and TAL_eed3_new (GTCCGTCACTCACTCTGTTGCTG) followed by a 2% agarose gel electrophoresis without enzymatic digestion.

To genotype paraformaldehyde-fixed embryos and larvae, DNA was extracted using sodium hydroxide and Tris [46]. Single embryos or larvae were placed into microcentrifuge tubes containing 20 μL 50 mM NaOH and heated 20 min at 95 °C. The tubes were then cooled to 4 °C, and 2 μL of 1 M Tris-HCl, pH 7.4, was added to neutralize the basic solution. Genotype analysis was performed on 2.5 μL of samples by PCR-BseNI digestion or PCR without digestion, as described above.

Sequence determination (GATC-Eurofin, Ebersberg, Germany) was performed after cloning of the PCR products into pCR-XL-TOPO (Invitrogen-ThermoFisher, Waltham, MA, USA) according to the manufacturer’s instructions.

### 2.5. Kaplan–Meier Analysis

For the establishment of survival curves, the tip of the caudal fin of embryos from an *eed^+/−^* incross was transected within the pigment gap distal to the circulating blood for genotyping purposes [46]. The embryos were then pooled according to their genotypes, placed into separate 1-L tanks, and incubated at 28 °C. Larvae were fed from 5 dpf on, 3 times per day, with early-stage zebrafish nutrition (Gemma Micro ZF75, Planktovie, France). The larvae were regularly examined directly in the tanks using a Leica EZ4 stereomicroscope with no or minimal manipulation during a period of 18 days after the spawn. Larvae were declared dead when heartbeats could not be detected. Dead larvae were immediately removed from the tanks.

### 2.6. Oil Red-O Staining

Zebrafish larvae were fixed overnight in 4% paraformaldehyde and pigments were bleached 2 h in 1% KOH, 3% H_2_O_2_. Larvae were stained with oil red-O (Hamiya Biomedical Company, Tukwila, WA, USA) for 15 min. Stained larvae were washed with PBS, stored in 70% glycerol, and imaged on a bright-field Leica MZ125 stereomicroscope equipped with a Leica DFC295 digital camera. After imaging, DNA was extracted using sodium hydroxide and Tris and genotyped.

### 2.7. Smurf Assay

Smurf assays were conducted according to Dambroise et al. [47] by placing zebrafish larvae in 50 mL Falcon tubes containing 2.5% blue #1 (erioglaucine disodium salt, Sigma) for 30 min at room temperature. They were then rinsed under aquarium water until no more blue coloration could be found in the eluate, anesthetized in 0.016% tricaine (MS-222; 3-amino benzoic acid ethyl ester, Sigma) and imaged on a bright-field stereomicroscope (Leica MZ125) equipped with a Leica DFC295 digital camera. After imaging, DNA was extracted for genotyping analyses.

### 2.8. Histological Analysis

For histological analyses, tail biopsies were used for genotyping before paraformaldehyde fixation of the larvae. Larvae were embedded in paraffin and cut into 5 μm-thick sections. These were mounted on sylanated glass slides, deparaffinated, rehydrated, and stained with hematoxylin and eosin for histological analysis as previously described [46].

### 2.9. Biometric Measurements

Standard length and eye diameter were measured according to Parichy et al. [48] on anesthetized larvae using ImageJ. After imaging and measurement, the larvae were genotyped.

Measurements of the surface of the pancreas labeled by in situ hybridization with a *prss1* probe were performed by equal intensity pixel counting using ImageJ.

Quantification of macrovesicular in the liver was performed on at least 3 consecutive hematoxylin and eosin-stained slides from 4 wild-type and 4 *eed^−/−^* mutant larvae using ImageJ. Each image was anonymized and transformed into an 8-bit grayscale picture before counting the hepatic nuclei and the number of vesicles.

For all biometric measurements, the software GraphPad Prism was used to build the graphs and to perform the statistical analyses.

### 2.10. Confocal Microscopy

For confocal imaging, tail biopsies were transected for genotyping before paraformaldehyde fixation of the larvae. After fixation in 4% paraformaldehyde, zebrafish larvae were maintained on glass-bottom dishes in 1.5% agarose. Fluorescent images were captured with an LSM 880 microscope and an AiryScan detector (ZEISS, Oberkoden, Germany). A Z-stack slice was made every 5 µm.

### 2.11. Whole-Mount In Situ Hybridization

Antisense-RNA probes were generated by RT-PCR from total mRNA extracted from zebrafish larvae at 5 dpf using the RNeasy Mini Kit (Qiagen, Courtaboeuf, France), following the manufacturer’s protocol. After reverse transcription (Superscript III, Invitrogen), cDNAs were amplified by PCR using the probe-specific primers, coupled to the T7 sequence for forward primers and the SP6 sequence for reverse primers. DIG-labeled antisense-RNA probes were synthetized using the DIG RNA Labeling Kit (SP6) (Roche–Sigma Aldrich Chimie S.a.r.l., Saint-Quentin-Follavier, France), following the manufacturer’s instructions.

The primers used for probe generation were:

ISHa_eed_F: TAATACGACTCACTATAGGG GCTGTGGCGGCTGGACTCTG

ISHa_eed_R: GATTTAGGTGACACTATAG CGCCAGATGGAGGCGTCGT

ISH_fabp2_F: TAATACGACTCACTATAGGG ATGACCTTCAACGGGACCTGG

ISH_fabp2_R: GATTTAGGTGACACTATAG AGCCCTCTTGAAAATCCTCTTGGC

ISH_ace2_F: TAATACGACTCACTATAGGGCTGTTGGAGAGATCATGTCGCTTTCT

ISH_ace2_R: GATTTAGGTGACACTATAGTGTCTTCCTCAAAGGCTTTGTTCACT

ISHa_fabp10a_F: TAATACGACTCACTATAGGG AAAGCATGGCCTTCAGCGGGA

ISHa_fabp10a_R: GATTTAGGTGACACTATAG GTCAGCGTCTCCACCATTTCTCCA

ISH_prss1_F: TAATACGACTCACTATAGGG TGCTCACTGCTACAAGTCCCGT

ISH_prss1_R: GATTTAGGTGACACTATAG CCCGAGCTTAGTTGGAGTTCATGGT

ISH_phox2bb_F: TAATACGACTCACTATAGGG GGGCCTAACCCGAACCCTACCTC

ISH_ phox2bb _R: GATTTAGGTGACACTATAG GAGCGCACATCGCAGTCTATCGG

ISH_pcna_F: TAATACGACTCACTATAGGG GGCAACATCAAGCTCTCACA

ISH_pcna_R: GATTTAGGTGACACTATAG AAATCCCACAGATGACAGGC

ISH_ccna2_F: TAATACGACTCACTATAGGGGGAAGGATGTCAACACAAGGAAG

ISH_ccna2_R: GATTTAGGTGACACTATAGGAGAGAACTGTCAGCACCAGATG

ISH_ccnd1_F: TAATACGACTCACTATAGGGTTTGCTGCGAAGTGGATACCA

ISH_ccnd1_R: GATTTAGGTGACACTATAGAACAGTTTGGGCGTGCTGAGT

ISH_sox2_F: TAATACGACTCACTATAGGG GTCCGAGAGCGAGAAGCGACC

ISH_sox2_R: GATTTAGGTGACACTATAGGCTGTAGGTGGGCGAGCCGT

ISH_mycn_F: TAATACGACTCACTATAGGGTGCGACTCCAAACGGGAGGCA

ISH_mycn_R: GATTTAGGTGACACTATAGGCTCACTTCGGGCGCTTTGACT

ISH_neurod1_F: TAATACGACTCACTATAGGGTCGAGACGCTCCGACTAGCCAA

ISH_neurod1_R: GATTTAGGTGACACTATAGGCGTCGAGCCCGCGTAAAGA

ISH_mag_F: TAATACGACTCACTATAGGGCCGTGAGGGTGTTCAGTGTGTGT

ISH_mag_R: GATTTAGGTGACACTATAGCGTCTCCCGTGCCTTCCTCT

RNA probes for *bmi1a* were synthesized with the DIG RNA Labeling Kit (SP6/T7) (Roche) on 1 μg of linearized cDNA clone MGC:56403 IMAGE:5605189 (imaGenes GmbH, Berlin, Germany).

Whole-mount in situ hybridizations were performed according to Thisse and Thisse [49]. Briefly, the fixed embryos were rehydrated and permeabilized with 10 µg/mL proteinase K for 30 sec (1-cell stage embryos), 10 min (24 hpf embryos), or 30 min (48 hpf embryos and 5 dpf larvae) at room temperature. Ten to 15 embryos from the TU strain of each time point, or about 50 larvae from *eed^+/−^* incrosses at 5 dpf, were hybridized with digoxigenin-labeled antisense-RNA probes at 70 °C. After extensive washing, the probes were detected with anti-digoxigenin-AP Fab fragment (Roche Diagnostics, 1093274, diluted at 1:10,000), followed by staining with BCIP/NBT (5-bromo-4-chloro-3-indolyl-phosphate/nitro blue tetrazolium) alkaline phosphate substrate. The embryos were imaged using a Leica MZ10F stereomicroscope equipped with a Leica DFC295 digital camera.

### 2.12. Histone Extraction and Western Blot Analysis

Histone extraction and Western blot analyses were performed from 10 larvae as described previously [46].

Primary antibodies used were mouse anti-H3K27me3 (1:1000; ab6002, Abcam, Paris, France), rabbit anti-H3K27me2 (1:500; ab24684, Abcam), rabbit anti-H3K27me1 (1:500; ab84932, Abcam), rabbit anti-H2AK119ub (1:2000; DC27C4; Cell Signaling), rabbit anti-H3K9me3 (1:1000; AB8898; Millipore-Sigma Aldrich S.a.r.l., Saint-Quentin-Follavier, France), rabbit anti-H4K20me3 (1:1000; ab9053; Abcam), rabbit anti-H3K27ac (1:1000; ab4729; Abcam) and rabbit anti-H3 (1:5000; ab1791, Abcam). The secondary antibodies were peroxidase conjugated anti-mouse antibody (1:10,000; 115-035-003, Jackson ImmunoResearch, Ely, UK) and peroxidase conjugated anti-rabbit antibody (1:10,000; 711-035-152, Jackson ImmunoResearch).

### 2.13. RNA Extraction, RT-PCR and RT-qPCR

RNA extraction and RT-PCR experiments were conducted as previously described [50]. cDNA was synthesized using Superscript III (18080-044, Invitrogen, Carlsbad, CA, USA) following the manufacturer’s instructions. A quantity of 1 µg of total RNAs were used to perform the reverse transcription experiments. Primers used were:

Dr_eed_cDNA_5′: GGAAACGAGATGCCGAACAA

Dr-eed_cDNA_3′a: CAGCCGGATCTCTCCCTGAG

Dr_cDNA_ube2a_F: TGACTGTTGACCCACCTTACAG

Dr_cDNA_ube2a_R: CAAATAAAAGCAAGTAACCCCG

Dr_beta-actin_cDNA_5′: CGTGACATCAAGGAGAAGCT

Dr_beta-actin_cDNA_3′: ATCCACATCTGCTGGAAGGT

qPCR_fasn_5′: CCAGCCATAAGAACGTCAGCCGAGA

qPCR_fasn_3′: CACCTTCCCGTCACACACCTCGT

qPCR_srebf1_5′: ACAGCGCGGCTAATGGCAGG

qPCR_srebf1_3′: TGCCCAGGAGCCGACAGGAA

qPCR_pparg_5′: GCACAGGCGCTTCAGTGTTCAG

qPCR_pparg_3′: CCAGCGAGTGCGTGTCGTCC

qPCR_nr1h3_5′: ACCCAGCGATTGACAGCATCACCT

qPCR_nr1h3_3′: CCTGCACGTTTGGTCGGTCTGCT

PCR reactions were performed as follow: 95 °C 4 min, (95 °C 45 s, 65 °C 45 s, 72 °C 1 min) 35 cycles, 72 °C 10 min.

The quantitative qPCR reaction was performed in triplicate using a QuantStudio 3 Real-Time System (Thermo Fisher Scientific, Waltham, MA, USA) using SYBR Green Supermix (Bio-Rad, Marne-la-Coquette, France). Relative mRNA expression of each gene was normalized to *ube2a* levels.

### 2.14. Behavioral Studies

Behavioral studies were performed on 5 dpf larvae from *eed^+/−^* incrosses in plates that were handled minimally before placement in a Zebrabox chamber (ViewPoint Life Sciences, Lyon, France) equipped with an infrared light-emitting floor and a top-mounted infrared camera allowing video recording of the whole plate under both light and dark conditions. Larval behavior measurements were achieved using the ZebraLab software with a detection threshold set at 35 and an xmin set at 3 (ViewPoint Life Sciences, Lyon, France). After recording, the larvae were euthanized, and their genotype was determined.

The locomotor activity assays were conducted in 48-well plates. The protocol consists of a 10 min initial acclimating period in the dark, followed by six alternating 10 min light and dark phases.

Thigmotaxis assays were conducted in 24-well plates. The timeline of the thigmotaxis assay protocol consists of a 6 min acclimatization step in the light followed by a 4 min dark challenge period. The arena was divided into two areas, inner and outer, of the equivalent surface. The diameter of the inner area corresponds to the total diameter of the well divided by $\sqrt{2}$. Thigmotaxis was presented as the percentage of the total distance moved or the total time spent by each larva into the outer zone during the challenge period.

## 3. Results

### 3.1. eed Expression during Zebrafish Development

**Figure 1:**
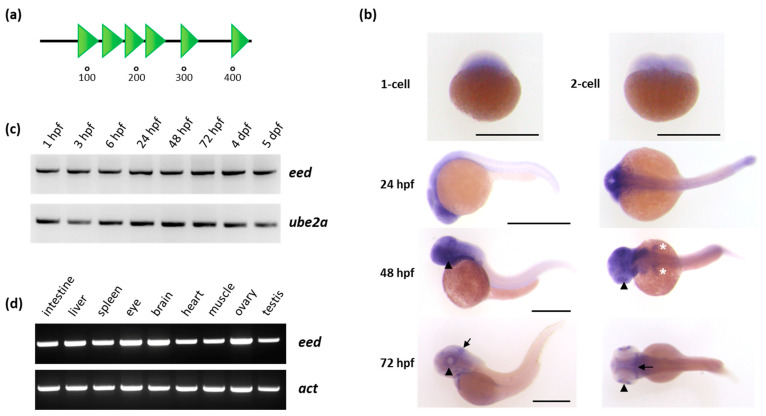
Organization of the Eed protein and *eed* mRNA expression in zebrafish: (**a**) schematic representation of the Eed protein. The zebrafish protein, as its human ortholog, is composed of 441 amino acids and contains six WD40 domains (SMART: SM000320) shown as green triangles; (**b**) whole-mount RNA in situ hybridization showing maternally provided *eed* transcripts at the 1-cell and 2-cell stage, and zygotic *eed* mRNA distribution at 24, 48 and 72 hpf. At these later stages, a lateral view is at the left and a dorsal view at the right. The arrowheads show the retina, the white asterisks the pectoral fin buds, and the arrow identify the midbrain-hindbrain boundary. Scale bar is 500 µm; (**c**) RT-PCR experiment showing the detection of *eed* transcripts at 1 hpf, 3 hpf, 6 hpf, 24 hpf, 48 hpf, 72 hpf, 4 dpf, and 5 dpf. *Ube2a* is used as a control; (**d**) RT-PCR experiment showing *eed* mRNAs in adult zebrafish tissues. Beta-actin (*act*) is used as a control. (See Supplementary Materials).

Human EED protein is encoded by 12 exons containing gene located on chromosome 11, upstream of the *HIKESHI* (*heat shock protein nuclear import factor hikeshi*) gene. The protein is composed of 441 amino acids and contains six WD40 domains adopting a β-propeller architecture forming multiple van der Waals interactions and hydrogen bonds with a domain located at the N-terminus of EZH2 [51]. EED is conserved across vertebrate species, both at the protein and genomic levels. In zebrafish, the *eed* gene is located on chromosome 1. It is organized in 12 exons positioned at the 5′ of the *hikeshi* gene, thus defining an evolutionarily conserved synteny block. The gene also codes for a 441-amino-acids-long protein sharing 91.4% identity with its human ortholog and containing six WD40 domains as well (Figure 1a).

In zebrafish, zygotic transcription is activated at about cell cycle 10–13, around 3.5 h post fertilization (hpf). Before this mid-blastula transition (MBT) stage, all developmental events depend on the maternally provided gene products [52,53]. Using in situ hybridization at 1-cell and 2-cell stages (Figure 1b) and RT-PCR at 1 and 3 hpf (Figure 1c), we found in agreement with previous studies [54,55] that *eed* mRNA is maternally loaded into the zebrafish embryo. After MBT, during tissue specification, *eed* zygotic expression is detected by RT-PCR (Figure 1c) and appears enriched in the anterior region of the embryo and in highly proliferative tissues such as the retina, the midbrain-hindbrain boundary, the pharyngeal arches, the pectoral fin buds or the intestine as shown by in situ hybridization at 24, 48 and 72 hpf (Figure 1b). Finally, in adult zebrafish, RT-PCR experiments showed that *eed* is ubiquitously expressed in all the tissues we tested (Figure 1d).

### 3.2. TALEN-Mediated Inactivation of eed in Zebrafish

**Figure 2:**
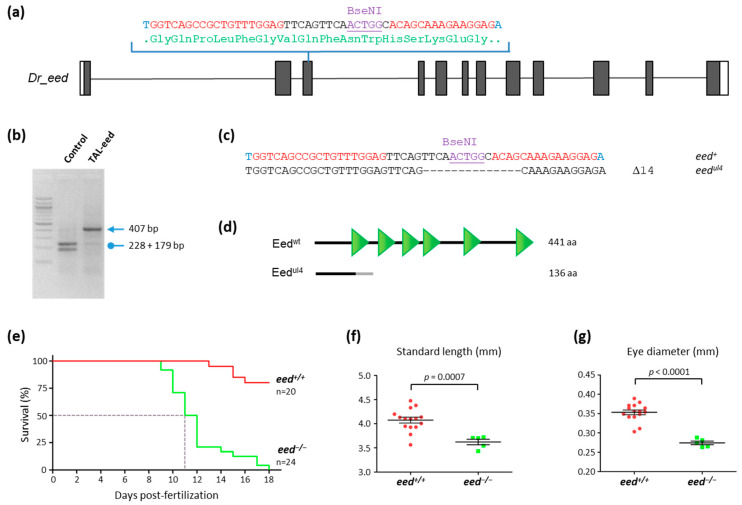
Loss of *eed* gene function leads to zebrafish death at around 10–12 dpf: (**a**) schematic representation of the genomic structure of the *eed* gene, with coding and untranslated sequences depicted as solid and open boxes, respectively. The sequence targeted by the TALEN in exon 3 is indicated with left and right TALEN binding sites shown in red, while the BseNI restriction sequence is underlined; (**b**) identification of mutant embryos using restriction fragment length polymorphism. Genomic DNA was prepared from an uninjected (Control) and an *eed* TALEN injected (TAL-eed) embryo, amplified by PCR and subjected to BseNI digestion. The TAL-eed injected embryo contains undigested material (arrow at 407 bp), indicating that the BseNI diagnostic restriction site has been disrupted; (**c**) sequence of the mutant allele compared to its wild-type counterpart. Dashes show deleted nucleotides. The mutated *eed^ul4^* allele possesses a deletion of 14 nucleotides; (**d**) schematic representation of wild-type (Eed^wt^) and predicted mutant (Eed^ul4^) proteins. The gray line in the predicted mutant protein corresponds to residues read out of frame prior to encountering a premature STOP codon. The green triangles in the wild-type protein show the WD40 domains; (**e**) Kaplan–Meier survival curves over 18 days for *eed^+/+^* (red curve) and *eed^−/−^* (green curve) siblings from a cross between heterozygous *eed^+/−^* fish. The number of fish considered is indicated; (**f**) measurement of the total length of *eed^+/+^* (red) and *eed^−/−^* (green) siblings from a cross between heterozygous *eed^+/−^* fish at 11 dpf. Statistical significance was assessed using a t-test; (**g**) measurement of the eye diameter of *eed^+/+^* (red) and *eed^−/−^* (green) siblings from a cross between heterozygous *eed^+/−^* fish at 11 dpf. Statistical significance was assessed using a Student *t*-test.

To generate *eed* mutations in zebrafish, we designed TALENs targeting the exon 3 within the sequence coding for the first WD40 repeat (Figure 2a). The targeted site was chosen to cover a BseNI restriction site used to screen for TALEN-induced mutations and for genotyping purposes (Figure 2b). Following TALEN mRNA injection in zebrafish embryos from the TU strain at the 1-cell stage, we isolated a line carrying an allele, *eed^ul4^*, with a deletion of 14 base pairs (Figure 2c). This mutation produces a frameshift leading to the synthesis of a predicted protein of 136 amino acids lacking all the WD40 domains (Figure 2d). Therefore, *eed^ul4^* is very likely a null allele and henceforth referred to as *eed^−^*.

Adult heterozygous *eed^+/−^* fish are viable, fertile, and do not show any obvious phenotype. In contrast, and similarly to *ezh2^−/−^* [37,38] and *suz12a^−/−^; suz12b^−/−^* fish [39], homozygous *eed^−/−^* mutants die at around 10–12 dpf (Figure 2e). Analysis of the living *eed^−/−^* mutants at 11 dpf showed that they do not present gross morphological alterations while they appear significantly smaller than wild-type siblings (Figure 2f,g). Then, *eed* function is not required for early development and normal formation of the body plan but is necessary for zebrafish survival after 10–12 dpf.

### 3.3. Loss of eed Function Alters H3K27me2/3 Levels, but Not H3K27me1 nor H2AK119ub

**Figure 3:**
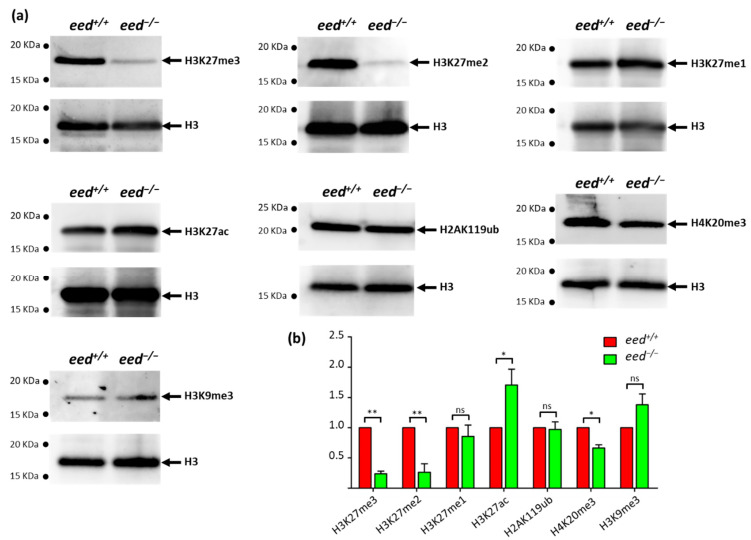
Effects of *eed* loss of function on global levels of histone modifications: (**a**) after genotyping of the caudal extremity of larvae, 10 to 15 µg of bulk histones from a pool of 10 *eed^+/+^* or *eed^−/−^* siblings at 9 dpf were analyzed by Western blot using specific antibodies recognizing H3K27me3, H3K27me2, H3K27me1, H2AK119ub, H3K27ac, H3K9me3 or H4K20me3. After stripping, the membranes were reprobed with an anti-histone H3 as a control; (**b**) comparison between *eed^+/+^* (red) and *eed^−/−^* (green) siblings, of histone modification signals normalized by total histone H3 and expressed relative to the levels found in the wild-type fish. Quantified levels are the mean ± SD of at least three independent histone extractions, except for H3K9me3, where two histone extraction were performed, followed by Western blot analysis. Statistical significance was assessed using a Student *t*-test; ns, non-significant; *, *p* < 0.05; **, *p* < 0.01. (See Supplementary Materials).

To investigate the effect of *eed* loss of function on histone modifications, we performed a series of Western blot analyses using antibodies recognizing specific histone marks (Figure 3). Histones were extracted from *eed^+/+^* and *eed^−/−^* siblings at 9 dpf and subjected to Western blot analyses to determine global levels of several histone modifications.

Not surprisingly, a great reduction in bulk H3K27me3 and H3K27me2 levels is observed in *eed^−/−^* homozygous larvae at 9 dpf. However, faint signals for H3K27me2/3 methylations are still detected in homozygous mutant fish, presumably due to the activity of the maternal products initially deposited in the embryos. In contrast, we did not find significant differences in H3K27me1 levels between wild-type and mutant larvae. This puzzling observation might suggest that in the absence of PRC2 activity, other histone lysine methyltransferases could monomethylate H3K27 and compensate for PRC2 loss of activity. Because mammalian G9a (EHMT2) and Glp (EHMT1) histone lysine methyltransferases have been shown to target H3K27 in vivo [56,57], their zebrafish orthologs (Ehmt1a, Ehmt1b, and Ehmt2) are potential candidates that could maintain H3K27me1 levels in the absence of PRC2 function. In agreement with other studies using knockout embryonic stem cells (ESCs) and in pathological situations where the loss of H3K27me2/3 correlates with increased acetylation at H3K27 [58,59], we also found that *eed^−/−^* homozygous larvae display a slight increase in global H3K27ac levels at 9 dpf (Figure 3).

Monoubiquitylation of H2AK119 contributes to polycomb repression and is achieved by the action of the catalytic subunits RING1/RNF2 of PRC1 [10,11]. PRC1 complexes consist of a variety of protein assemblies with diverse compositions and are classified as canonical (cPRC1) and non-canonical (ncPRC1) complexes [60,61]. This classification is mainly based on the presence of a chromobox (CBX) protein in cPRC1 complexes and YY1-binding protein (RYBP), or its homolog YAF2, in ncPRC1 complexes. Whereas cPRC1 is recruited to H3K27me3 via its CBX subunit, ncPRC1 complexes mediate H2A ubiquitylation at polycomb target sites independently of PRC2 action and of the presence of H3K27me3 epigenetic marks [60,61,62]. The examination of bulk H2AK119ub did not show differences between *eed^−/−^* and *eed^+/+^* homozygous larvae at 9 dpf, indicating that in the absence of PRC2 function, ncPRC1 complexes are able to maintain normal levels of H2AK119ub (Figure 3).

Finally, to check whether an increase in the heterochromatin marks H3K9me3 and H4K20me3 could compensate for the decrease in H3K27me2/3, we investigated their relative abundance in *eed^−/−^* homozygous mutant larvae at 9 dpf, compared to wild-type. If a decrease in H4K20me3 levels could be found in PRC2-deficient fish, we were not able to detect significant differences in bulk H3K9me3 (Figure 3). Then, the normal formation of the body plan in *eed^−/−^* mutants cannot be explained by the compensation of H3K27me2/3 loss through the increase in other repressive marks.

### 3.4. Loss of eed Function Alters the Homeostasis of Digestive Organs in Zebrafish

**Figure 4:**
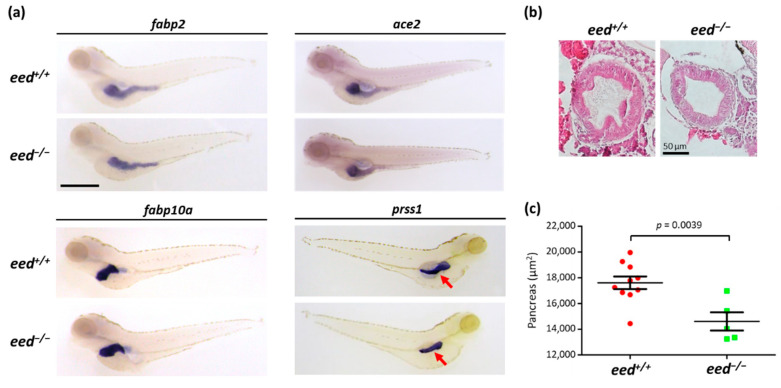
Organization of the digestive organs at 5 dpf: (**a**) whole-mount RNA in situ hybridization to detect the expression of markers of the intestine (*fabp2*, *ace2*), the liver (*fapb10a*), and the exocrine pancreas (*prss1*) in *eed^+/+^* and *eed^−/−^* siblings at 5 dpf. The red arrow shows the pancreas. Scale bar is 500 µm; (**b**) intestinal bulb sections from *eed^+/+^* (left) and *eed^−/−^* (right) larvae at 5 dpf stained with hematoxylin and eosin. Scale bar is 50 µm; (**c**) measurement of the surface of the pancreas labeled by in situ hybridization using a *prss1* probe at 5 dpf for *eed^+/+^* (red) and *eed^−/−^* (green) larvae. Statistical significance was assessed by a Student t-test, and the corresponding *p*-value is indicated.

Because it has been shown that *ezh2*-deficient zebrafish develop intestinal defects [38,63], we investigated the potential effect of *eed* loss of function on the development of digestive organs. First, we examined the expression of markers of the intestine (*fabp2*, fatty acid-binding protein 2, intestinal and *ace2*, angiotensin I converting enzyme 2), the liver (*fabp10a*, fatty acid-binding protein 10a, liver basic), and the exocrine pancreas (*prss1*, serine protease 1 also known as trypsin, *try*) in 5-dpf old larvae by in situ hybridization (Figure 4a). Pools of larvae from heterozygous *eed^+/−^* incrosses were subjected to in situ hybridization and individually pictured before being genotyped by RFLP.

The expression of *fabp2*, *ace2,* and *fabp10a* revealed that the intestine and the liver are normally developed in *eed^−/−^* larvae at 5 dpf. Furthermore, histological studies showed that the structure of the intestine is identical in *eed^+/+^* and *eed^−/−^* zebrafish at this stage (Figure 4b). In contrast, and similar to what was observed in *ezh2^−/−^* mutants [38], the size of the pancreas appears smaller in *eed*-deficient fish at 5 dpf (Figure 4a,c).

**Figure 5:**
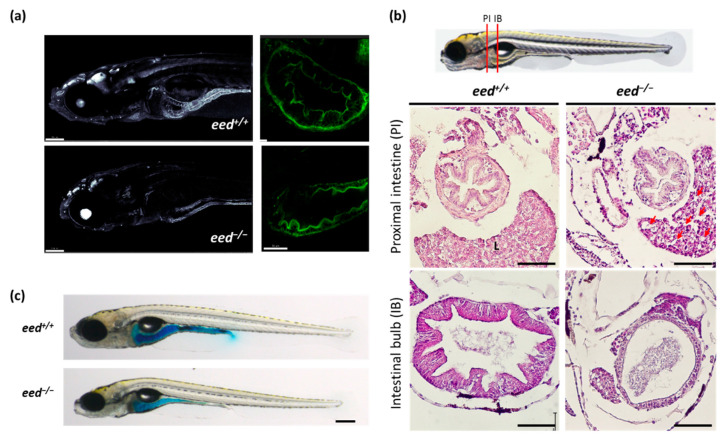
Structure of the intestine at 9–11 dpf: (**a**). confocal imaging of the anterior part (left, scale bar is 150 µm) and of the intestine bulb (right, scale bar is 50 µm) of transgenic Tg (actb2:GFP-Hsa.UTRN)^e116^ zebrafish larvae, wild-type (up) or lacking *eed* function (down) at 9 dpf; (**b**) histological sections stained with hematoxylin and eosin at the levels of the proximal intestine (PI) and the intestinal bulb (IB) as indicated from *eed^+/+^* (left) and *eed^−/−^* (right) siblings at 11 dpf. Red arrows show macrovesicles. L, liver. Scale bar is 50 µm; (**c**) Smurf assay performed on *eed^+/+^* and *eed^−/−^* siblings at 11 dpf. Scale bar is 200 µm.

To visualize the intestine structure in the whole larvae at a later developmental stage by confocal microscopy, we used the transgenic line Tg(actb2:GFP-Hsa.UTRN)^e116^ in which actin is labeled by the green fluorescent protein (GFP) [40,64,65]. Fluorescence imaging revealed an alteration of the intestine structure of the *eed^−/−^* transgenics at 9 dpf. Indeed, the number of cells forming the thickness of the intestinal wall and visible through GFP expression is decreased in *eed*-deficient zebrafish as of 9 dpf (Figure 5a). Then, while the intestine develops normally until 5 dpf, its integrity fails to be maintained at later developmental stages.

The analysis of histological sections of larvae at 11 dpf confirmed that the intestine wall of *eed^−/−^* mutants is strongly reduced and lacks folds at the level of the intestinal bulb (Figure 5b). By contrast, the structure of the proximal intestine does not show these strong defects, as previously observed in *ezh2^−/−^* mutants [38]. Because the intestine looks normal at 5 dpf but altered at 9–11 dpf, we conclude that in the absence of *eed* and PRC2 functions, the development of the intestine occurs, but the integrity of its structure is not maintained. To investigate the permeability of the intestine in mutants, we performed a Smurf assay [47]. In this assay, the permeability of the intestine was assessed by evaluating the diffusion throughout the body of blue food dye present in the fish water. Figure 5c shows that in spite of a strong alteration of the structure of the intestine bulb, the intestinal permeability is not increased.

**Figure 6:**
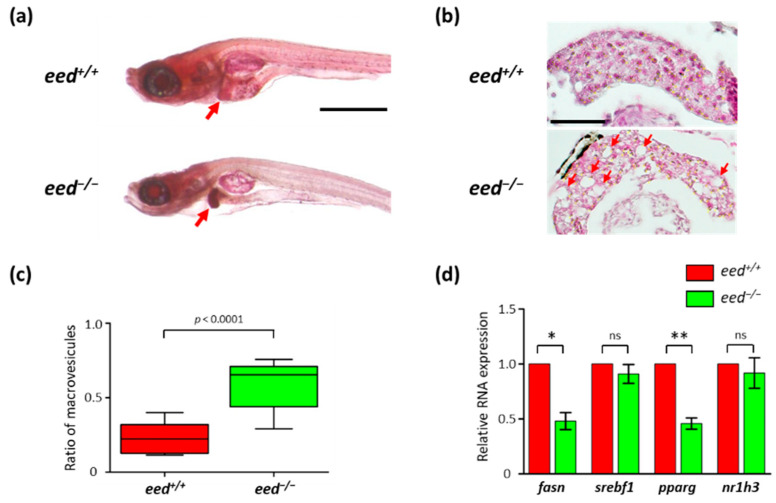
Liver alterations in *eed*-deficient larvae: (**a**) representative images of lateral views of *eed^+/+^* and *eed^−/−^* larvae stained with oil red-O at 11 dpf. The red arrow shows the liver. Scale bar is 500 µm; (**b**) histological sections stained with hematoxylin and eosin showing the liver of *eed^+/+^* and *eed^−/−^* larvae at 11 dpf. Red arrows show macrovesicles. Scale bar is 50 µm; (**c**) quantification of macrovesicular steatosis *eed^+/+^* (red) and *eed^−/−^* (green) larvae at 11 dpf. The ratio of macrovesicular structures relative to total hepatic surface was calculated on 3 histological slices per larvae from 4 different larvae per genotype. Statistical significance was assessed using a Mann–Whitney *t*-test; (**d**) relative levels of *fasn*, *srebf1*, *pparg,* and *nr1h3* RNA abundances in 9 dpf old *eed^+/+^* (red) and *eed^−/−^* (green) larvae quantified by RT-qPCR. Three independent experiments were performed, and error bars represent standard deviation. Statistical analysis was performed using a one-way ANOVA test followed by a Dunn’s multiple test comparison; ns, non-significant; *, *p* < 0.05; **, *p* < 0.01.

Neutral lipid dye oil red-O staining of larval zebrafish is commonly used to examine steatosis caused by consumption of a high-fat diet or induced by various drugs [66,67,68,69,70]. Oil red-O staining clearly shows a strong accumulation of neutral lipids in the liver of *eed^−/−^* larvae à 11 dpf when compared to wild-type larvae (Figure 6a). Such a strong oil red-O staining of the liver was also found in *ezh2^−/−^* homozygous mutants [38], whereas exposure to Ezh1/2 inhibitor PF-06726304 acetate increases lipid accumulation in larval zebrafish [71]. Moreover, hematoxylin and eosin stain on histological sections of zebrafish larvae at 11 dpf showed signs of macrovesicular steatosis in *eed^−/−^* larvae (Figure 5b and Figure 6b,c).

To investigate the impact of *eed* loss of function on the expression of lipogenic factors, we performed RT-qPCRs to determine RNA abundance for *fasn* (fatty acid synthetase), *srebf1* (sterol regulatory element-binding transcription factor 1), *pparg* (peroxisome proliferator-activated receptor gamma), and *nr1h3* (nuclear receptor subfamily 1, group H, member 3, also known as liver X receptor *lxr*) at 9 dpf. Then, in spite of showing an increase in lipid accumulation, RNA levels for lipogenic factors remain either unchanged (*srebf1* and *nr1h3*) or reduced (*fasn* and *pparg*) in *eed^−/−^* larvae (Figure 6d). This suggests that *eed* loss of function-induced steatosis is likely the result of an increase in lipid storage in the liver rather than an increase in lipid synthesis.

Taken together, our study of *eed*-deficient zebrafish mutants demonstrates that PRC2 loss of function has various effects on the homeostasis of digestive organs, ranging from a delay in pancreatic development, a loss of maintenance of the intestine structures to hepatic steatosis.

### 3.5. Loss of eed Function Alters Neuronal Differentiation

**Figure 7:**
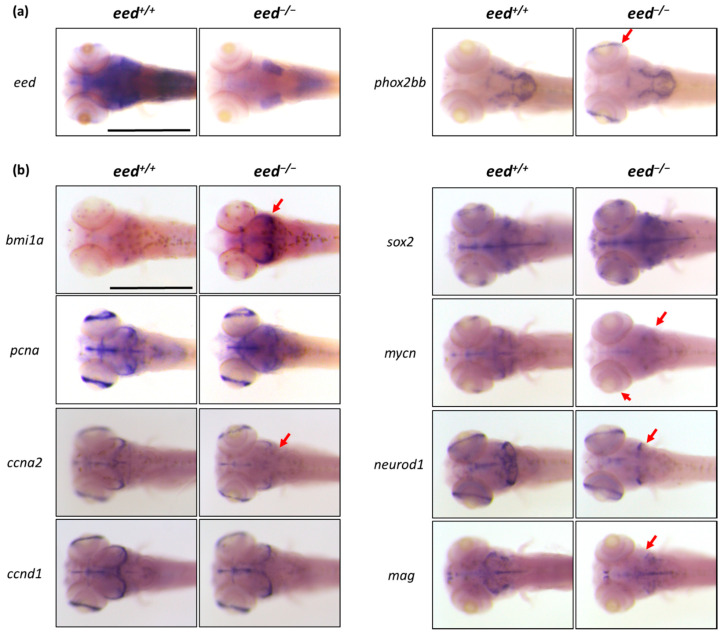
Whole-mount RNA in situ hybridization of the brain region of *eed^+/+^* and *eed^−/−^* siblings at 5 dpf: (**a**) whole-mount in situ hybridization to detect *eed* (left) and *phox2bb* RNA expression. The red arrow shows the *phox2bb* ectopic expression in the retina of *eed* mutants. Scale bar is 500 µm; (**b**) whole-mount in situ hybridization to detect the expression profiles of stemness (*bmi1a*, *sox2*), proliferation (*pcna*, *mycn*), cell cycle (*ccna2*, *ccnd1*), and neuronal/glial precursor (*neurod1*, *mag*) markers. The red arrows emphasize expression profile differences between *eed^+/+^* and *eed^−/−^* larvae. Scale bar is 500 µm.

To characterize in more detail our *eed*-deficient zebrafish mutants, we performed additional whole-mount RNA in situ hybridization experiments at 5 dpf with a focus on the brain. Mutant and wild-type siblings were treated in one batch for each probe and documented photographically. The genotype of each embryo was then determined by RFLP on genomic DNA extracted from the fixed and stained material. First, we showed that *eed* expression is strongly reduced in *eed^−/−^* larvae, presumably due to nonsense-mediated decay (NMD) of the mutant transcript (Figure 7a). Second, we investigated the expression profile of *phox2bb* (*paired like homeobox 2Bb*), a transcription factor expressed by a subset of cells in the posterior regions of the brain. In *eed^−/−^* mutants, *phox2bb* appears additionally expressed ectopically in the retina (Figure 7a). This *phox2bb* misexpression in the retina was also observed in *ezh2*-deficient zebrafish larvae [38]. Then, *eed* loss of function recapitulates the defects found in *ezh2^−/−^* mutants at the level of the digestive organs, but also in terms of *phox2bb* expression in the brain and the retina. Moreover, while *eed* is expressed in large territories of the brain, its loss of function impairs *phox2bb* specifically in the retina, indicating that PRC2 controls gene expression programs in a very subtle and discreet manner, depending on the cell type.

To gain insights into the possible role of PRC2 in brain development, we applied in situ hybridizations at 5 dpf on several genes known to be expressed in the midbrain-hindbrain boundary region that contains neural stem cells and dividing progenitors [72], and where *eed* transcripts are abundant (Figure 1a and Figure 7a) [54]. The genes chosen for the study were the stemness markers *bmi1a* (*bmi1 polycomb ring finger oncogene 1a*) and *sox2* (SRY-box transcription factor 2); the proliferation marker *pcna* (proliferating cell nuclear antigen); *mycn* (MYCN proto-oncogene, bHLH transcription factor), which is involved in several signaling pathways promoting cell growth, proliferation and metabolism of progenitor cells in different developing organs and tissues; two genes coding for cyclins, *ccna2* (cyclin A2) and *ccnd1* (cyclin D1); *neurod1* (*neuronal differentiation 1*), a marker of neuronal precursors; and *mag* (myelin-associated glycoprotein), a marker of glial precursors (Figure 7b).

Analysis of expression profiles of the stemness markers *bmi1a* and *sox2* revealed a strong increase in *bmi1a* labeling at the midbrain-hindbrain boundary of *eed^−/−^* mutants suggesting that the number of stem cells is increased, possibly due to differentiation defects in this region. In contrast, *sox2* expression seems less affected by the loss of *eed* function, with eventually a slight increase in *sox2* RNA abundance in the hindbrain. The RNA expression profile of *pcna* appeared globally similar in *eed^+/+^* and *eed^−/−^* larvae indicating that PRC2 loss of function has little or no effect on cell proliferation in the developing brain at 5 dpf. However, while expression of the cyclin gene *ccnd1* is unchanged in *eed^−/−^* larvae, transcription of *ccna2* appears in part slightly deregulated. We observed that the expression of *mycn* is downregulated in the hindbrain and the retina of the *eed* mutants. Finally, using probes for neuronal precursors (*neurod1*) and glial precursors (*mag*), whole-mount RNA in situ hybridizations showed a reduction in the labeled areas indicating that the numbers of these precursors are diminished in the hindbrain or midbrain of *eed^−/−^* larvae, respectively. Altogether, our results suggest that PRC2 loss of function disturbs the differentiation and the development of a subset of neurogenic cells in the midbrain-hindbrain region and in the retina without dramatically affecting cell proliferation, as judged by *pcna* expression. Thus, remarkably, while *eed* loss of function drastically affects *bmi1a* expression, the effect of the mutation on the expression of other markers such as *ccna2*, *mycn*, *neurod1,* or *mag* remains relatively thin and affects discrete areas of the brain.

### 3.6. Loss of eed Function Alters Locomotor Activity

Loss of *eed* function alters the differentiation of neurogenic cells at 5 dpf. Then, to investigate whether *eed* deficiency could also affect larval behavior, we performed locomotor and thigmotaxis assays. Zebrafish larvae from heterozygous *eed^+/−^* incrosses were individually disposed of into dish wells. The plates were handled minimally before placement in a Zebrabox chamber (ViewPoint Life Sciences, Lyon, France) equipped with an infrared light-emitting floor and a top-mounted infrared camera allowing video recording of the whole plate under both light and dark conditions. Larval behavior measurements were achieved using the ZebraLab software (Version 5.18.0.0, ViewPoint Life Sciences, Lyon, France). After recording, the larvae were euthanized and their genotype determined by RFLP.

**Figure 8:**
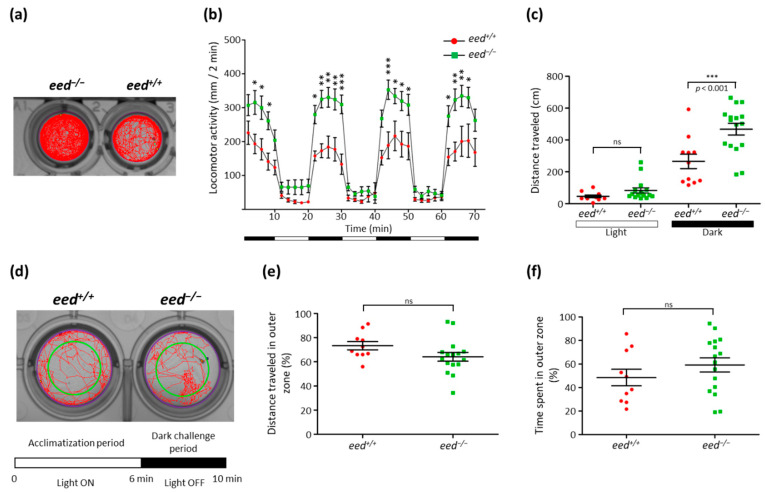
Behavioral comparison between wild-type and *eed^−/−^* mutants at 5 dpf: (**a**) locomotor tracking for a 70 min recording of an *eed^−/−^* mutant (left) and a wild-type (right) larvae; (**b**) distance traveled throughout a 70 min session for wild-type (red, *n* = 11) and *eed^−/−^* mutant (green, *n* = 16). Data are presented as mean ± SD of the distance moved (in mm) in 2 min intervals. Black and white bars at the bottom indicate dark and light conditions, respectively. Statistical analysis was performed using two-way ANOVA followed by Bonferroni posttest comparisons; ns, non-significant; *, *p* < 0.05; **, *p* < 0.01; ***, *p* < 0.001; (**c**) cumulative distance traveled for each wild-type (red) and mutant (green) larvae during the light (left) and dark (right) periods. Statistical analysis was performed using one-way ANOVA followed by Bonferroni posttest comparisons; ns, non-significant; ***, *p* < 0.001; (**d**) the experimental procedure for the thigmotaxis assay is composed of a 6 min acclimatization period with the light ON followed by a 4 min visual motor challenge period with the lights OFF. Experiments are performed in a 24-well plate format. Inner and outer zones are delineated by the green circle in a way that the two zones cover equivalent spatial areas. The distance traveled during the dark challenge period is shown for two larvae; (**e**) cumulative distance traveled in the outer zone of wild-type (red, *n* = 10) and *eed^−/−^* mutant (green, *n* = 16) larvae. Each point represents individual larvae. No statistical (ns) difference was found between wild-type and *eed^−/−^* mutants; (**f**) cumulative time spent in the outer zone of wild-type (red, *n* = 10) and *eed^−/−^* mutant (green, *n* = 16) larvae. Each point represents unique larvae. No statistical (ns) difference was found between wild-type and *eed^−/−^* mutants.

The locomotor activity assays were conducted in 48-well plates following a protocol that consists of a 10 min initial acclimating period in the dark, followed by six alternating 10 min light and dark phases (Figure 8a). As previously described [73], switching to light dramatically decreases larval activity, whereas return to darkness is associated with an increase in locomotor activity. Additional cycles of alternating light and dark produce alternating levels of low and high activity of the zebrafish larvae, respectively (Figure 8b). Behaviorally, *eed^−/−^* larvae are hyperactive compared with wild-type larvae at 5 dpf. This hyperactive phenotype of *eed^−/−^* mutant is particularly prominent and significant during the dark cycles (Figure 8b,c).

Thigmotaxis (or “wall hugging”) is a common behavioral endpoint used in preclinical studies employing rodents but also applied to the zebrafish model [74,75]. Animals engaged in thigmotaxic behavior strongly avoid the center of an arena and reduce their exploration behavior to stay or move in close proximity to the boundaries of the environment. Thigmotaxis assays were conducted in 24-well plates using a protocol composed of a 6 min acclimatization period in the light followed by a 4 min dark challenge period. The arena was divided into two areas, inner and outer, of equivalent surface, and thigmotaxis was presented as the percentage of the total distance moved or the total time spent by each larva into the outer zone (Figure 8d). Our measurements showed that there is no significant difference in thigmotaxis behavior between *eed^−/−^* mutants and wild-type larvae at 5 dpf (Figure 8e,f).

Thus, in spite of having a hyperactive phenotype, the *eed^−/−^* mutants do not present an increase in their exploration behavior when compared to wild-type siblings.

## 4. Discussion

PRC2 is a histone-modifying protein complex conserved from fruit flies to humans and able to deposit H3K27me1/2/3 epigenetic marks responsible in turn to local chromatin compaction and gene repression. Genome-wide studies using human or mouse embryonic stem cells showed that PRC2 and H3K27me3 marks are enriched at promoters of numerous developmental genes, leading to the proposal that PRC2 could be involved in the maintenance of the pluripotency of stem cells by keeping developmental genes repressed [12,13,14,15,16,17,18,19]. Differentiation is then associated with the relocation of the PRC2 complexes turning stem cell genes expression off and switching on gene programs specific to given developmental lineages. PRC2 is composed of the core subunit EZH1/2, EED, SUZ12, and RBBP4/7 together with a wide range of substoichiometric subunits defining distinct alternate protein complexes [22]. However, PRC2 catalytic activity relies only on the presence of the two mutually exclusive histone methyltransferases EZH1 or EZH2, associated with EED and SUZ12. In zebrafish, as the result of a genome duplication that occurred in the teleost lineage, the Suz12 subunit is encoded by two ohnologues, *suz12a* and *suz12b*. Then, *eed* is the sole zebrafish gene coding for a PRC2 subunit present in all alternate complexes. Consequently, since EED is required for EZH1/2 methyltransferase activity, it is expected that inactivation of the zebrafish *eed* gene will result in the loss of activity of all PRC2 complexes.

Using the TALEN technology, we generated an *eed* allele harboring a 14 bp deletion within the third exon. This deletion conducts to a frameshift within the coding sequence leading to the production of a putative protein lacking all the WD40 domains, predicted to be non-functional, and therefore likely producing an *eed* null allele. Heterozygous *eed^+/−^* fish are viable, fertile, and do not present visible gross phenotypes. By opposition, *eed^−^* homozygosity leads to larval death at around 12 dpf. This time point is similar to what was observed for zebrafish lacking the function of other PRC2 components, such as for *ezh2* mutants [37,38] or *suz12a; suz12b* double-mutants [39], but contrasts to *Eed* knockout mouse phenotype [25]. While mouse embryos homozygous for the *Eed* mutation fail to gastrulate normally, *eed* embryonic expression is not required for the establishment of the zebrafish body plan. This relatively late lethality makes the zebrafish a compelling model to study PRC2 function during the development of various tissues without the requirement of conditional knockout strategies. It also reinforces the idea that PRC2 function plays little or no role in the implementation of developmental decisions but is mainly required to maintain these developmental instructions.

Zebrafish *eed^−/−^* mutants display various alterations of the digestive organs. These defects include a smaller pancreas at 5 dpf, hepatic steatosis characterized by an increase in lipids and macrovesicles in the liver, and the loss of maintenance of the intestine structures particularly marked at the level of the intestinal bulb. Studies in mice also pointed out the role of *Eed* in intestinal development [76]. Indeed, the conditional knockout of *Eed* in postnatal intestinal crypts decreases the stem cell pools, reduces the proliferation potential, increases the secretory cell differentiation, and results in smaller mouse intestines. Zebrafish larvae hatch with a yolk sac, which is depleted at 6–7 dpf. Then, after that time, their growth relies on external food uptake. If left unfed, the larvae will usually die of starvation at around 12 dpf [77]. Thus, it is very likely that the defects of the digestive organs found in the *eed^−/−^* homozygous mutants account for their larval death at 12 dpf. Furthermore, the marked *eed^−/−^* phenotype at the level of the digestive organs closely resembles the phenotype of the *ezh2^−/−^* homozygous mutants we have previously described [38]. These indistinguishable phenotypes exclude critical roles of Ezh1 and/or PRC2-independent Ezh2 functions in the development of the digestive organs, at least until 12 dpf.

Investigations on bulk histone modifications in *eed*-deficient larvae at 9 dpf revealed a dramatic decrease in H3K27me2/3 marks, as expected. However, *eed^−/−^* larvae retained some H3K27me2 and H3K27me3 residual methylation marks. We assume that the remaining H3K27me2/3 signals were deposited by the maternal PRC2 activity and retained in slowly dividing or quiescent cells. Maternal PRC2 contribution was previously demonstrated by showing that maternal-zygotic *MZezh2^−/−^* mutants display a much stronger phenotype than zygotic *ezh2^−/−^* mutants, with small eyes, accumulation of blood near the yolk extension, stringy heart, heart edema, and absence of pectoral fins at 2 dpf [37]. EZH1 and EZH2 are both able to deposit H3K27me1, but the role of PRC2 in the control of H3K27me1 monomethylation is still a matter of debate [20,58,78,79]. Studies using *Eed* and *Suz12* knockout mouse ESC lines, as well as knockdown experiments in mouse ESCs, showed that the inhibition of PRC2 activity is responsible for a global loss of H3K27me2 and H3K27me3 levels, but at best for a partial reduction in H3K27me1 marks [24,80,81]. Our analysis in zebrafish revealed that H3K27me1 global levels are not significantly changed in *eed^−/−^* larvae indicating that in the absence of PRC2 function, H3K27 monomethylation is achieved by other histone methylation routes. It has been shown that the histone lysine methyltransferases G9a (EHMT2) and Glp (EHMT1), in addition to being potent H3K9 methyltransferases, are able to monomethylate H3K27 both in vitro and in mouse ESCs [56,57,82]. Thus, their zebrafish orthologs, Ehmt1a, Ehmt1b, and Ehmt2, appear as suitable candidates to account for monomethylation at H3K27 targets in the absence of PRC2 activity.

Polycomb repression is achieved by the combined action of both PRC2 and PRC1. While PRC2 methylates H3K27, PRC1 monoubiquitylates H2AK119. Multiple PRC1 subcomplexes have been shown to coexist in cells. These complexes are commonly referred to as canonical (cPRC1) or non-canonical (ncPRC1), based on their dependence on H3K27me3 recruitment to target loci [6,83]. We have shown that global H2AK119ub levels were unchanged in *eed^−/−^* larvae indicating H2K119 monoubiquitylation is maintained in the absence of PRC2 function, presumably thanks to ncPRC1 action. This situation is similar to what was observed in ESCs, where Ezh1/Ezh2 double knockout was reported to do not affect H2K119ub levels [58]. H2K119ub maintenance in *eed^−/−^* mutants also explains the milder phenotype observed in *eed*-deficient fish when compared to *rnf2^−/−^* mutants. In zebrafish, Rnf2 is the only catalytic subunit common to all PRC1 subcomplexes [84]. Zebrafish deficient in *rnf2* gene function lack H2K119ub marks, display several defects including jaw malformation, pericardial edema, diminished blood circulation, absence of pectoral fins, severe craniofacial phenotype, and die at around 4–5 dpf [85,86]. However, our results contrast with another study using maternal-zygotic *MZezh2* zebrafish mutants [87]. In this report, it was shown that loss of Ezh2 function from maternal and zygotic origins resulted in dramatic impairment of PRC1 recruitment at the chromatin and complete loss of H3K119ub marks. This observation highlighted the role of the PRC2 in the functional recruitment of cPRC1 complexes and led us to propose that in the absence of zygotic Eed function, maternal PRC2 activity is sufficient to deposit enough H3K27me3 marks allowing cPRC1 recruitment at early developmental stages. Then, at later stages, H2AK119ub marks are propagated and transmitted through cell generations in a PRC2-independent fashion via the action of ncPRC1 complexes. This hypothesis would explain why *rnf2^−/−^* and *MZezh2^−/−^* mutants display more severe phenotypes than *ezh2* and *eed* homozygous zygotic mutants do. Furthermore, it has been demonstrated that ncPRC1 complexes are able to propagate H2AK119ub during cell division via their recruitment to the chromatin through the binding of the RYBP/YAF2 subunits to H2AK119ub marks [88].

Sequencing studies on pediatric high-grade gliomas, including glioblastoma multiformes (GBMs) and diffuse intrinsic pontine gliomas (DIPGs), identify recurrent somatic lysine to methionine substitutions at position 27 (K27M) in histone H3. The H3^K27M^ mutation primarily occurs in *H3F3A* (>70%), one of the two genes coding for the histone variant H3.3, with a lower frequency in *HIST1H3B* and *HIST1H3C* encoding H3.1, and more rarely in *HIST2H3C* coding for H3.2 [89,90,91,92]. The H3.3^K27M^ mutated histone binds to EZH2 and inhibits the methyltransferase activity of the PRC2, leading to a dramatic decrease in global H3K27me3 marks and alterations in gene expression programs [93,94,95,96,97]. These changes in gene expression would sustain the undifferentiated phenotype of the tumor cells that contributes to the aggressiveness of the gliomas and to the dismal associated prognosis [94]. In addition to *H3F3A^K27M^* mutations, DIPGs also contain genetic alterations targeting canonical signaling pathways, most frequently *TP53* loss of function (about 60%) and gain-of-function mutations or amplifications in platelet-derived growth factor receptor alpha (*PDGFRA*; about 40%) [89,98], as well as numerous other mutations that might contribute to significant inter- and intratumoral DIPG heterogeneity and found at lower-frequencies [99]. However, the individual contribution of each of the different mutations, including *H3F3A^K27M^*, in glioma genesis remains poorly understood. In this context, we took advantage of our *eed*-deficient zebrafish line to investigate the effects of PRC2 loss of activity on neuronal differentiation and proliferation. We found that in *eed^−/−^* larvae, expression of some stemness (*bmi1a*), as well as neuronal (*neurod1*) or glial (*mag*) precursor markers, is altered, indicating that neural differentiation is impaired in the absence of functional PRC2 activity. Interestingly, a number of human DIPGs have been shown positive for BMI1 expression, which then appears as a potential therapeutic target [100,101,102]. By contrast, our in situ hybridization experiments revealed that the *pcna* expression profile remained unchanged in the brain of *eed^−/−^* larvae suggesting that PRC2 loss of function and reduction in H3K27me3 marks do not affect the proliferation potential of neural cells. Thus, we propose that in DIPGs, the *H3F3A^K27M^* mutation is responsible for the maintenance of an undifferentiated state, whereas other mutations might contribute to proliferation.

Surprisingly, we also showed that while expressed ubiquitously in the brain of wild-type larvae, *eed* loss of function results in gene expression alterations in relatively thin and discrete areas of the brain. For instance, ectopic expression of *phox2bb* is specifically found in the retina, whereas *neurod1* expression is lost in a subset part of the hindbrain. Then, the effects of PRC2 loss of function appear strictly cell-specific and context-dependent, differentially affecting cell fates. These subtle changes in gene expression would indubitably escape to global transcriptomic analyses. Thus, our observations also outline the importance of performing whole-mount in situ hybridizations, but not only global transcriptomic analyses, for the characterization of zebrafish lines harboring mutations in polycomb group genes. In this context, it is worth emphasizing that the loss of *eed* function gives different phenotypes depending on the considered organ. It affects neuronal differentiation in specific brain regions, the development of the pancreas, the maintenance of the intestine wall structures, or the metabolism of lipids in the liver. This variation in effects and phenotypes might reflect the fact that Eed controls different gene expression programs in the different organs. It also reinforces the idea that a better deciphering of PRC2 function would rely on transcriptomic analyses at the single-cell level.

Cohen-Gibson syndrome (COGIS, OMIM #617561) is an overgrowth disorder characterized by dysmorphic facial features, advanced bone age, skeletal abnormalities, associated with variable intellectual disability, and caused by de novo mutations in EED [30,103]. To date, COGIS (EED-related overgrowth) has been reported in eight individuals, with at least one patient showing hyperactivity [104]. Interestingly, our behavioral studies revealed that *eed^−/−^* larvae also present a hyperactive phenotype, raising the possibility that the *eed*-deficient zebrafish line could constitute a pertinent model to study the neurological defects leading to behavioral disabilities in COGIS.

## Data Availability

All relevant data are within the manuscript.

## Supplementary Materials

The following are available online at https://www.mdpi.com/article/10.3390/cells10113142/s1, Figure S1: Uncropped RT-PRC gels used in Figure 1; Figure S2: Uncropped Western blot used in Figure 3.
