# Supplementary material for: Loss of Polycomb Repressive Complex 2 Function Alters Digestive Organ Homeostasis and Neuronal Differentiation in Zebrafish
Source: Cells. 2021 Nov 12;10(11):3142. doi: 10.3390/cells10113142 (PMC8620594; doi:10.3390/cells10113142)

Figure S1: Uncropped RT-PRC gels used in Figure 1

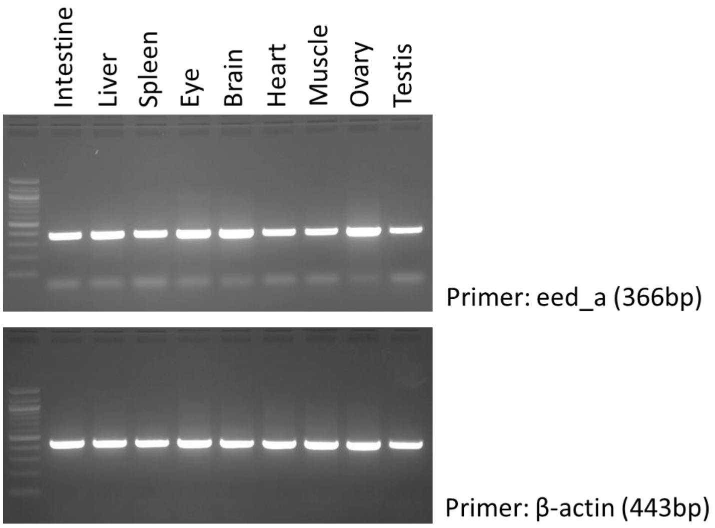

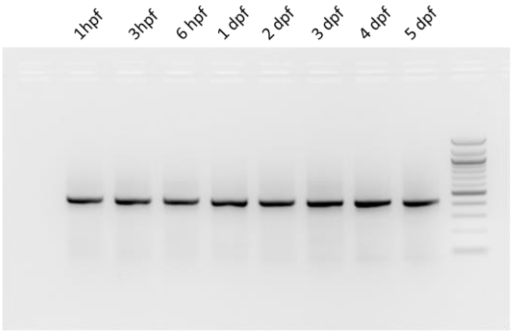

PCR product eed: 419bp
Dr_eed_cDNA_5 GGAAACGAGATGCCGAACAA
Dr_eed_cDNA_3 CACGCACACGTGTAGAAGTT

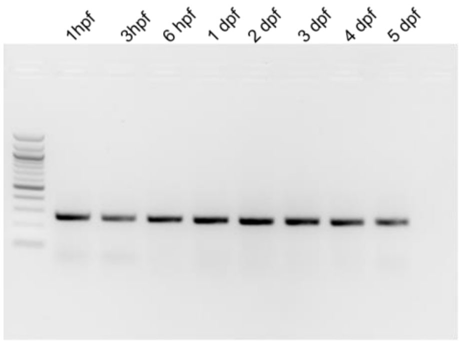

PCR product ube2a: 265bp

Dr_cDNA_ube2a_F TGACTGTTGACCCACCTTACAG
Dr_cDNA_ube2a_R CAAATAAAAGCAAGTAACCCCG

Figure S2: Uncropped Western blots used in Figure 3. After genotyping of the caudal extremity of larvae, 10 to 15 µg of bulk histones from a pool of 10 *eed*$^{+/+}$ or *eed*$^{-/-}$ siblings at 9 dpf were analyzed by Western blot using specific antibodies recognizing H3K27me3, H3K27me2, H3K27me1, H2AK119ub, H3K27ac, H3K9me3 or H4K20me3 (Blots at the left side). After stripping, the membranes were reprobed with an anti-histone H3 as a control (Blots at the left side).

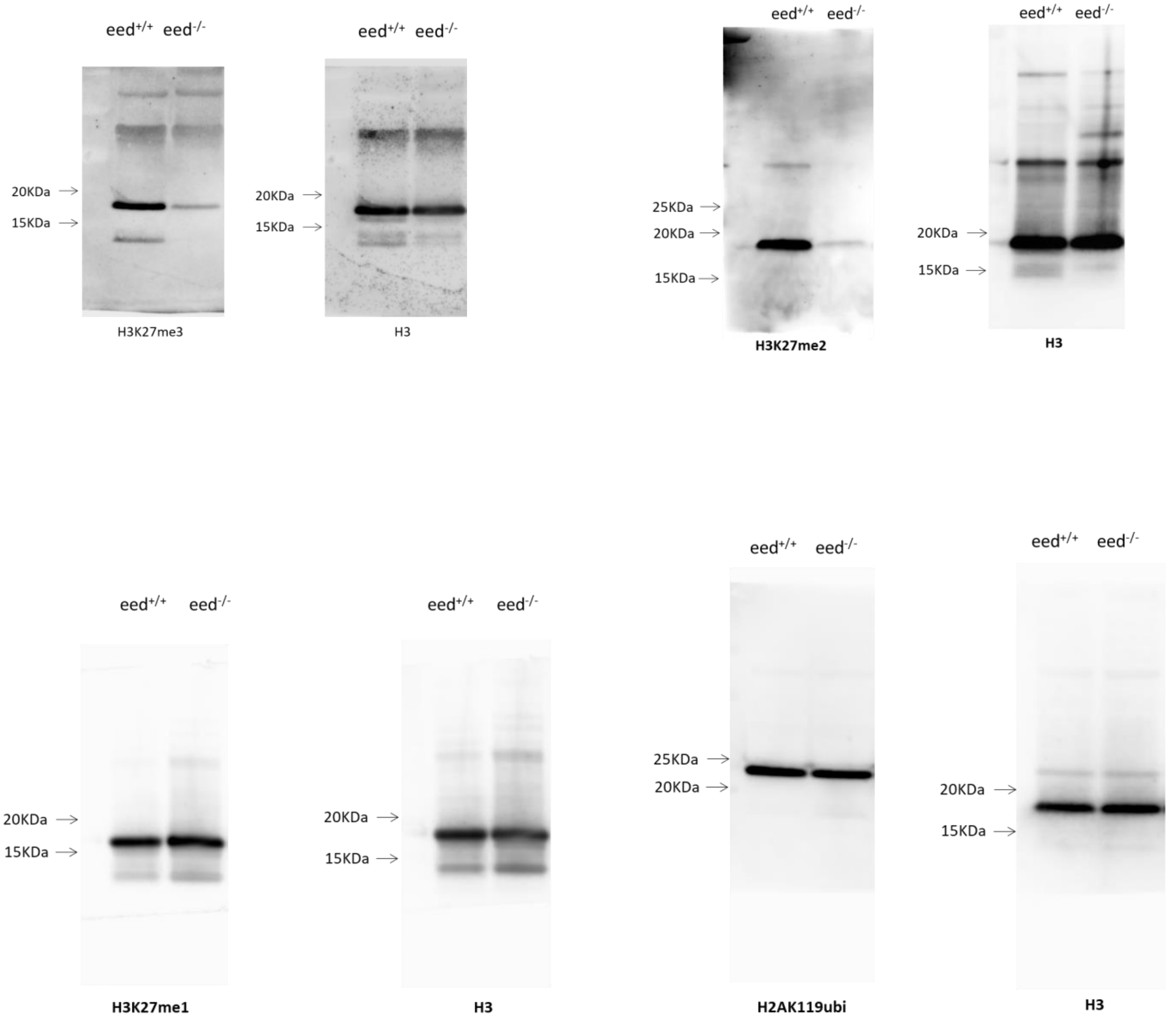

Figure S2: Uncropped Western blots used in Figure 3 (continued)

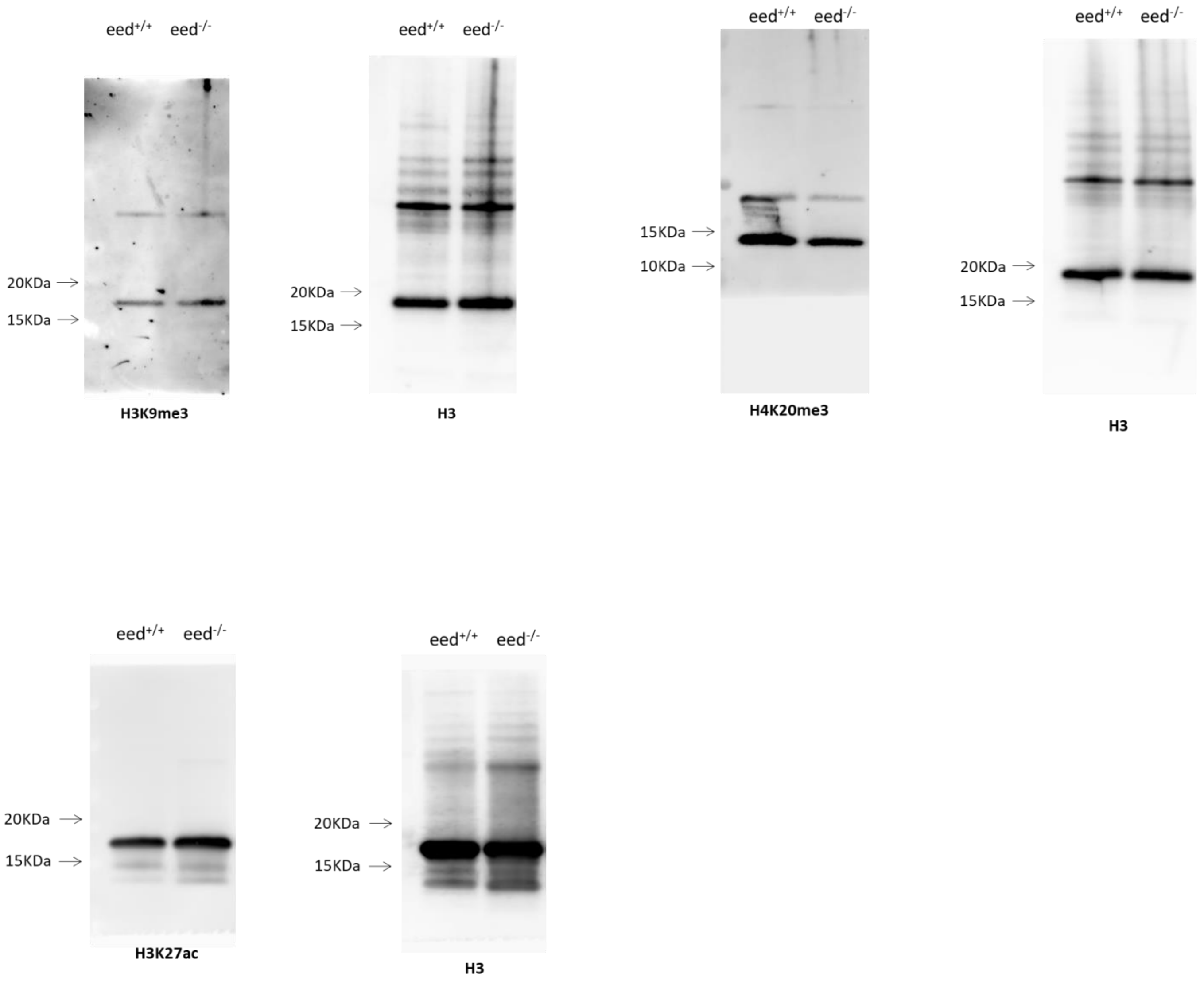

Supplement: Supplementary file 1 [file cells-10-03142-s001.zip › cells-1438961-supplementary.pdf]
